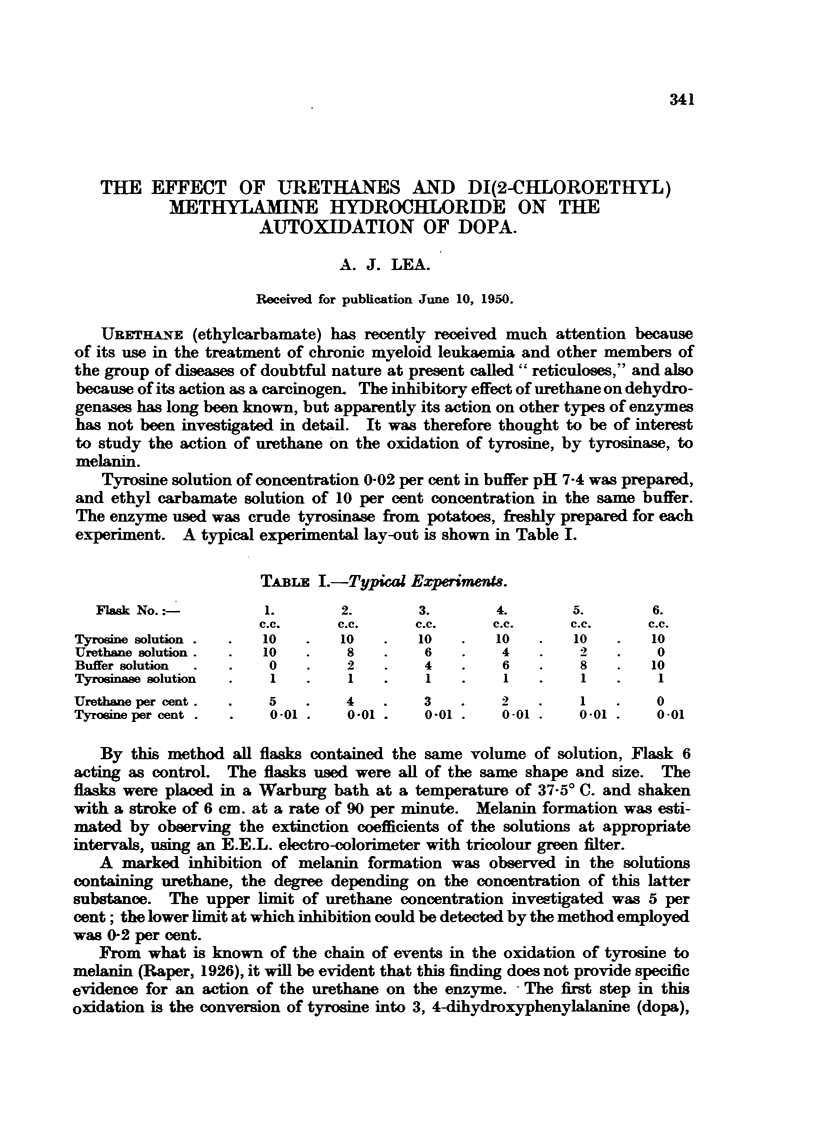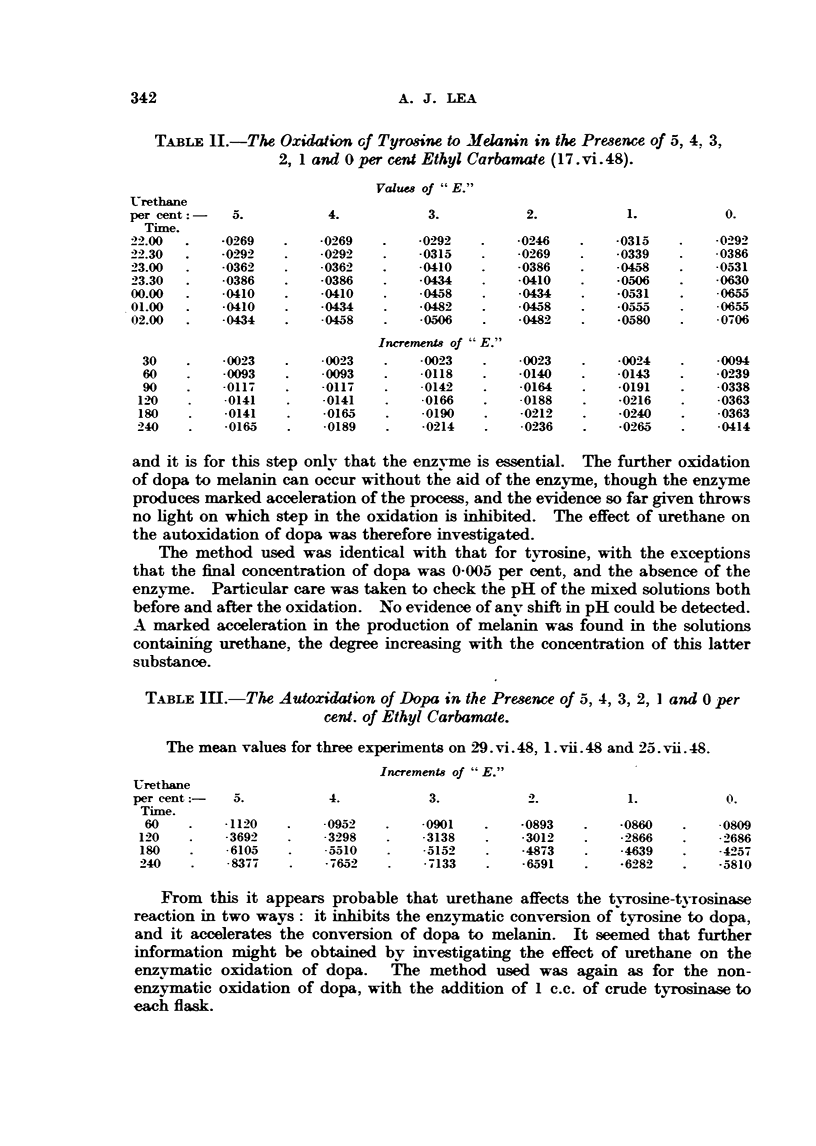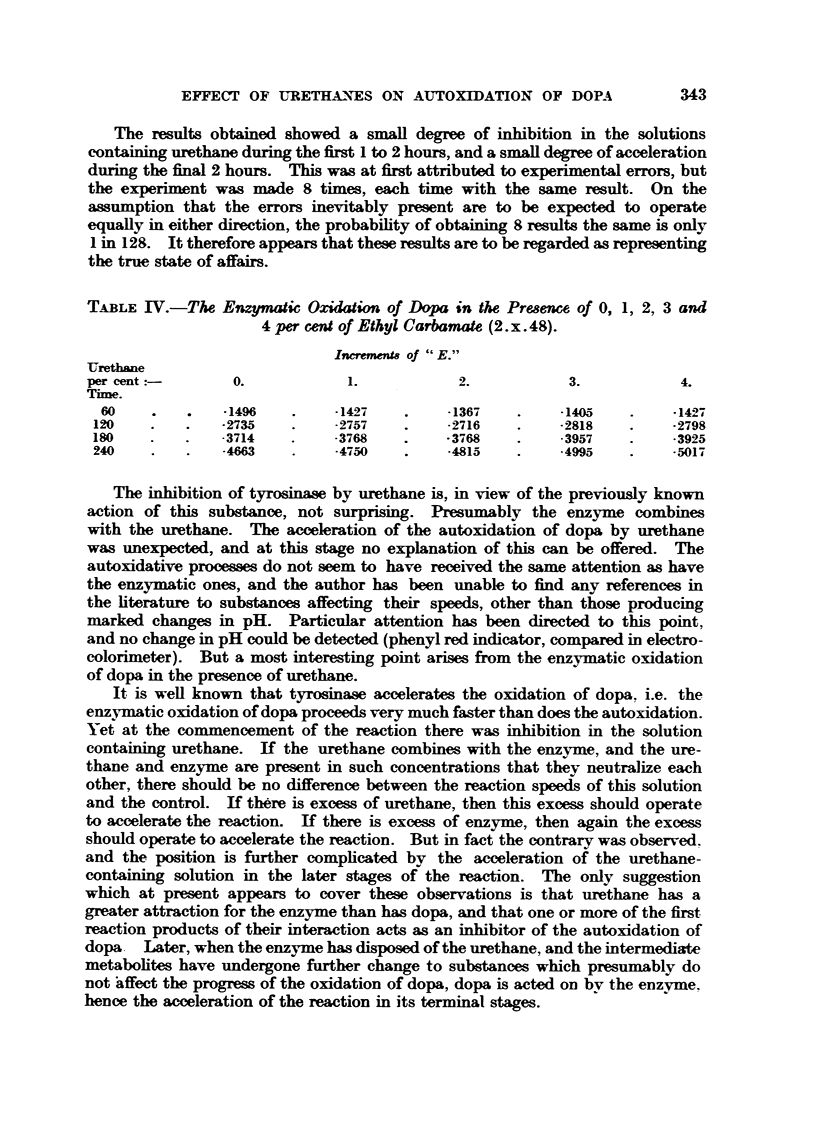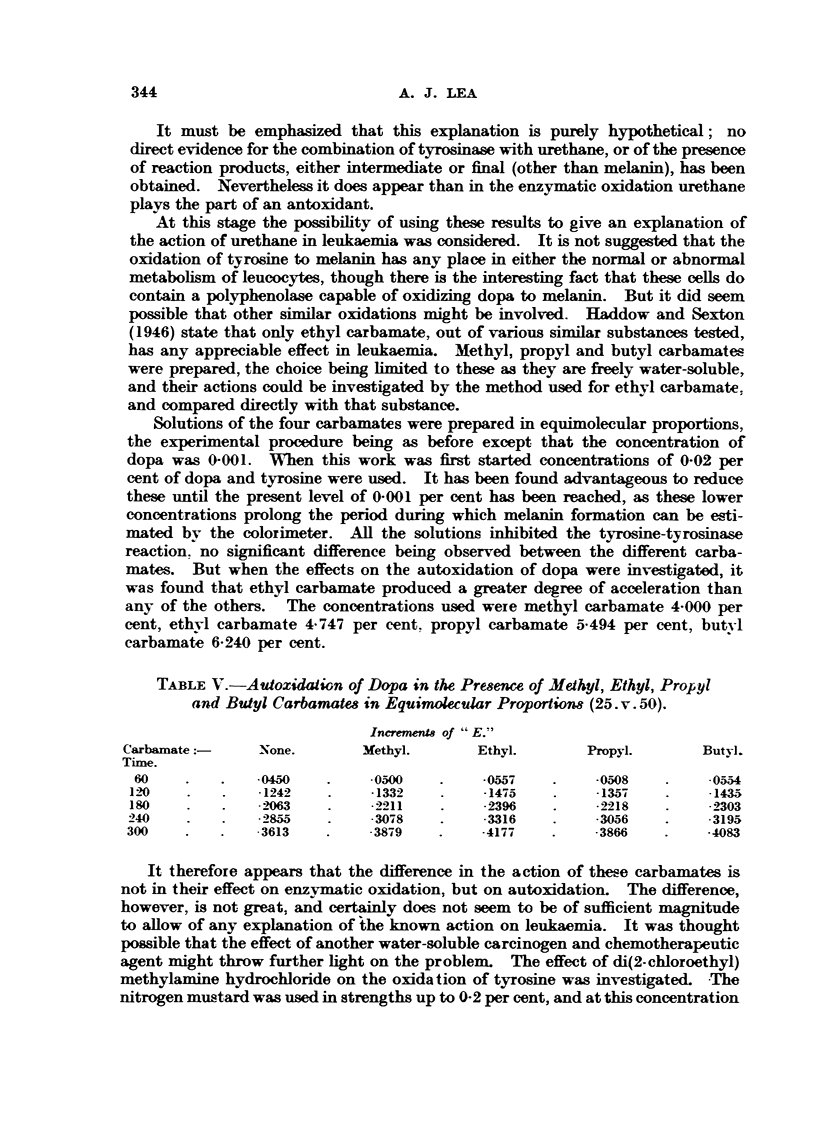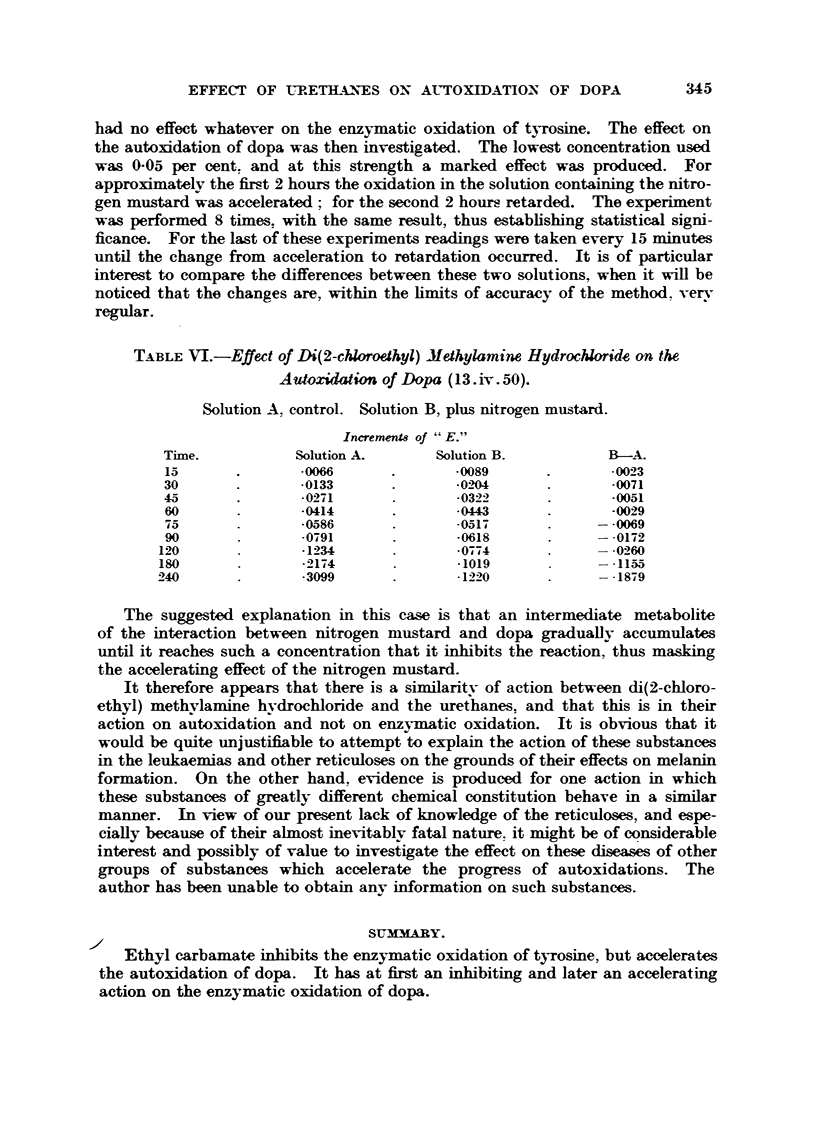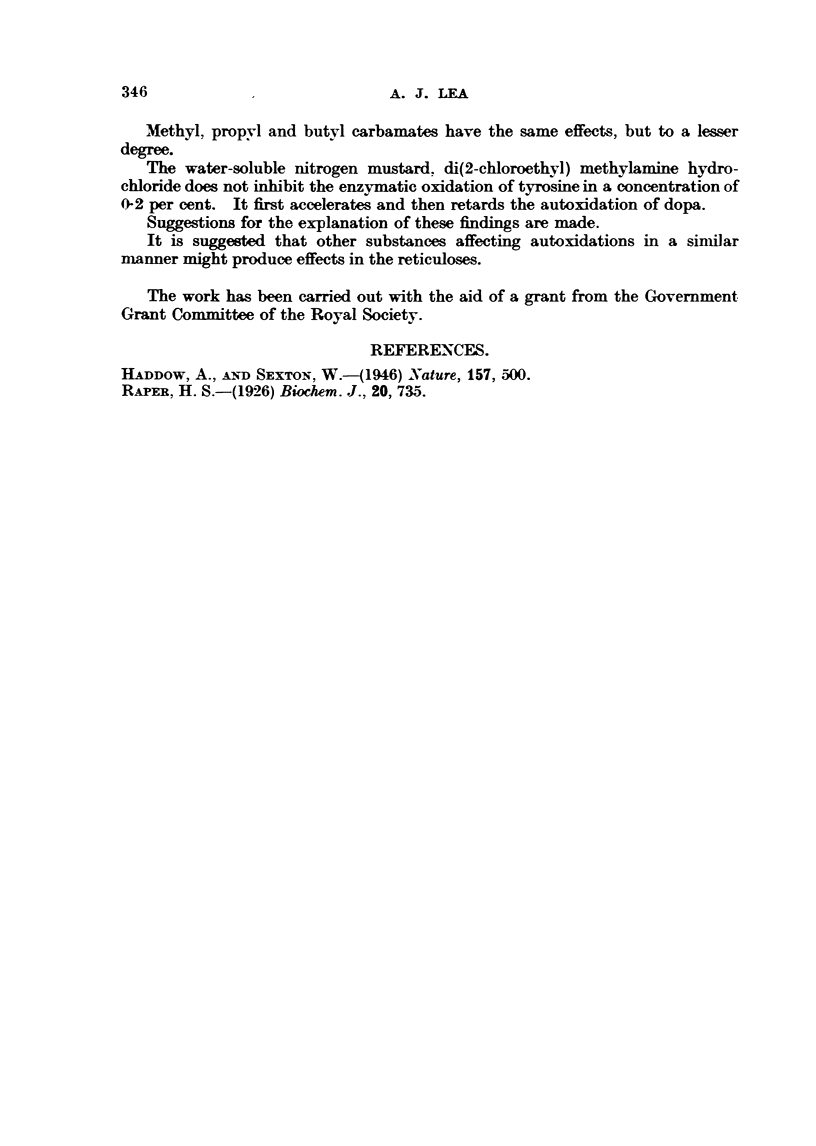# The Effect of Urethanes and Di(2-chloroethyl) Methylamine Hydrochloride on the Autoxidation of Dopa

**DOI:** 10.1038/bjc.1950.33

**Published:** 1950-09

**Authors:** A. J. Lea


### THE EFFECT OF URETHANES AND DI(2-CHLOROETHYL) METHYLAMINE HYDROCHLORIDE ON THE AUTOXIDATION OF DOPA.

A. J. LEA.

Received for publication June 10, 1950.

URETHANE (ethylcarbamate) has recently received much attention because of its use in the treatment of chronic myeloid leukaemia and other members of the group of diseases of doubtful nature at present called "reticuloses," and also because of its action as a carcinogen. The inhibitory effect of urethane on dehydrogenases has long been known, but apparently its action on other types of enzymes has not been investigated in detail. It was therefore thought to be of interest to study the action of urethane on the oxidation of tyrosine, by tyrosinase, to melanin.

Tyrosine solution of concentration 0·02 per cent in buffer pH 7·4 was prepared, and ethyl carbamate solution of 10 per cent concentration in the same buffer. The enzyme used was crude tyrosinase from potatoes, freshly prepared for each experiment. A typical experimental lay-out is shown in Table I.

TABLE I.—*Typical Experiments.*

| Flask No. :— | 1. | 2. | 3. | 4. | 5. | 6. |
|---|---|---|---|---|---|---|
| | c.c. | c.c. | c.c. | c.c. | c.c. | c.c. |
| Tyrosine solution | 10 | 10 | 10 | 10 | 10 | 10 |
| Urethane solution | 10 | 8 | 6 | 4 | 2 | 0 |
| Buffer solution | 0 | 2 | 4 | 6 | 8 | 10 |
| Tyrosinase solution | 1 | 1 | 1 | 1 | 1 | 1 |
| Urethane per cent | 5 | 4 | 3 | 2 | 1 | 0 |
| Tyrosine per cent | 0·01 | 0·01 | 0·01 | 0·01 | 0·01 | 0·01 |

By this method all flasks contained the same volume of solution, Flask 6 acting as control. The flasks used were all of the same shape and size. The flasks were placed in a Warburg bath at a temperature of 37·5° C. and shaken with a stroke of 6 cm. at a rate of 90 per minute. Melanin formation was estimated by observing the extinction coefficients of the solutions at appropriate intervals, using an E.E.L. electro-colorimeter with tricolour green filter.

A marked inhibition of melanin formation was observed in the solutions containing urethane, the degree depending on the concentration of this latter substance. The upper limit of urethane concentration investigated was 5 per cent; the lower limit at which inhibition could be detected by the method employed was 0·2 per cent.

From what is known of the chain of events in the oxidation of tyrosine to melanin (Raper, 1926), it will be evident that this finding does not provide specific evidence for an action of the urethane on the enzyme. The first step in this oxidation is the conversion of tyrosine into 3, 4-dihydroxyphenylalanine (dopa),

TABLE II.—*The Oxidation of Tyrosine to Melanin in the Presence of* 5, 4, 3, 2, 1 *and* 0 *per cent Ethyl Carbamate* (17.vi.48).

| Urethane per cent :— Time. | 5. | 4. | 3. | 2. | 1. | 0. |
|---|---|---|---|---|---|---|
| | | | *Values of "E."* | | | |
| 22.00 | ·0269 | ·0269 | ·0292 | ·0246 | ·0315 | ·0292 |
| 22.30 | ·0292 | ·0292 | ·0315 | ·0269 | ·0339 | ·0386 |
| 23.00 | ·0362 | ·0362 | ·0410 | ·0386 | ·0458 | ·0531 |
| 23.30 | ·0386 | ·0386 | ·0434 | ·0410 | ·0506 | ·0630 |
| 00.00 | ·0410 | ·0410 | ·0458 | ·0434 | ·0531 | ·0655 |
| 01.00 | ·0410 | ·0434 | ·0482 | ·0458 | ·0555 | ·0655 |
| 02.00 | ·0434 | ·0458 | ·0506 | ·0482 | ·0580 | ·0706 |
| | | | *Increments of "E."* | | | |
| 30 | ·0023 | ·0023 | ·0023 | ·0023 | ·0024 | ·0094 |
| 60 | ·0093 | ·0093 | ·0118 | ·0140 | ·0143 | ·0239 |
| 90 | ·0117 | ·0117 | ·0142 | ·0164 | ·0191 | ·0338 |
| 120 | ·0141 | ·0141 | ·0166 | ·0188 | ·0216 | ·0363 |
| 180 | ·0141 | ·0165 | ·0190 | ·0212 | ·0240 | ·0363 |
| 240 | ·0165 | ·0189 | ·0214 | ·0236 | ·0265 | ·0414 |

and it is for this step only that the enzyme is essential. The further oxidation of dopa to melanin can occur without the aid of the enzyme, though the enzyme produces marked acceleration of the process, and the evidence so far given throws no light on which step in the oxidation is inhibited. The effect of urethane on the autoxidation of dopa was therefore investigated.

The method used was identical with that for tyrosine, with the exceptions that the final concentration of dopa was 0·005 per cent, and the absence of the enzyme. Particular care was taken to check the pH of the mixed solutions both before and after the oxidation. No evidence of any shift in pH could be detected. A marked acceleration in the production of melanin was found in the solutions containing urethane, the degree increasing with the concentration of this latter substance.

TABLE III.—*The Autoxidation of Dopa in the Presence of* 5, 4, 3, 2, 1 *and* 0 *per cent. of Ethyl Carbamate.*

The mean values for three experiments on 29.vi.48, 1.vii.48 and 25.vii.48.

| Urethane per cent :— Time. | 5. | 4. | 3. | 2. | 1. | 0. |
|---|---|---|---|---|---|---|
| | | | *Increments of "E."* | | | |
| 60 | ·1120 | ·0952 | ·0901 | ·0893 | ·0860 | ·0809 |
| 120 | ·3692 | ·3298 | ·3138 | ·3012 | ·2866 | ·2686 |
| 180 | ·6105 | ·5510 | ·5152 | ·4873 | ·4639 | ·4257 |
| 240 | ·8377 | ·7652 | ·7133 | ·6591 | ·6282 | ·5810 |

From this it appears probable that urethane affects the tyrosine-tyrosinase reaction in two ways : it inhibits the enzymatic conversion of tyrosine to dopa, and it accelerates the conversion of dopa to melanin. It seemed that further information might be obtained by investigating the effect of urethane on the enzymatic oxidation of dopa. The method used was again as for the non-enzymatic oxidation of dopa, with the addition of 1 c.c. of crude tyrosinase to each flask.

The results obtained showed a small degree of inhibition in the solutions containing urethane during the first 1 to 2 hours, and a small degree of acceleration during the final 2 hours. This was at first attributed to experimental errors, but the experiment was made 8 times, each time with the same result. On the assumption that the errors inevitably present are to be expected to operate equally in either direction, the probability of obtaining 8 results the same is only 1 in 128. It therefore appears that these results are to be regarded as representing the true state of affairs.

TABLE IV.—*The Enzymatic Oxidation of Dopa in the Presence of* 0, 1, 2, 3 *and* 4 *per cent of Ethyl Carbamate* (2.x.48).

| | *Increments of "E."* | | | | |
|---|---|---|---|---|---|
| Urethane per cent :— Time. | 0. | 1. | 2. | 3. | 4. |
| 60 | ·1496 | ·1427 | ·1367 | ·1405 | ·1427 |
| 120 | ·2735 | ·2757 | ·2716 | ·2818 | ·2798 |
| 180 | ·3714 | ·3768 | ·3768 | ·3957 | ·3925 |
| 240 | ·4663 | ·4750 | ·4815 | ·4995 | ·5017 |

The inhibition of tyrosinase by urethane is, in view of the previously known action of this substance, not surprising. Presumably the enzyme combines with the urethane. The acceleration of the autoxidation of dopa by urethane was unexpected, and at this stage no explanation of this can be offered. The autoxidative processes do not seem to have received the same attention as have the enzymatic ones, and the author has been unable to find any references in the literature to substances affecting their speeds, other than those producing marked changes in pH. Particular attention has been directed to this point, and no change in pH could be detected (phenyl red indicator, compared in electro-colorimeter). But a most interesting point arises from the enzymatic oxidation of dopa in the presence of urethane.

It is well known that tyrosinase accelerates the oxidation of dopa, i.e. the enzymatic oxidation of dopa proceeds very much faster than does the autoxidation. Yet at the commencement of the reaction there was inhibition in the solution containing urethane. If the urethane combines with the enzyme, and the urethane and enzyme are present in such concentrations that they neutralize each other, there should be no difference between the reaction speeds of this solution and the control. If there is excess of urethane, then this excess should operate to accelerate the reaction. If there is excess of enzyme, then again the excess should operate to accelerate the reaction. But in fact the contrary was observed, and the position is further complicated by the acceleration of the urethane-containing solution in the later stages of the reaction. The only suggestion which at present appears to cover these observations is that urethane has a greater attraction for the enzyme than has dopa, and that one or more of the first reaction products of their interaction acts as an inhibitor of the autoxidation of dopa. Later, when the enzyme has disposed of the urethane, and the intermediate metabolites have undergone further change to substances which presumably do not affect the progress of the oxidation of dopa, dopa is acted on by the enzyme, hence the acceleration of the reaction in its terminal stages.

It must be emphasized that this explanation is purely hypothetical; no direct evidence for the combination of tyrosinase with urethane, or of the presence of reaction products, either intermediate or final (other than melanin), has been obtained. Nevertheless it does appear than in the enzymatic oxidation urethane plays the part of an antoxidant.

At this stage the possibility of using these results to give an explanation of the action of urethane in leukaemia was considered. It is not suggested that the oxidation of tyrosine to melanin has any place in either the normal or abnormal metabolism of leucocytes, though there is the interesting fact that these cells do contain a polyphenolase capable of oxidizing dopa to melanin. But it did seem possible that other similar oxidations might be involved. Haddow and Sexton (1946) state that only ethyl carbamate, out of various similar substances tested, has any appreciable effect in leukaemia. Methyl, propyl and butyl carbamates were prepared, the choice being limited to these as they are freely water-soluble, and their actions could be investigated by the method used for ethyl carbamate, and compared directly with that substance.

Solutions of the four carbamates were prepared in equimolecular proportions, the experimental procedure being as before except that the concentration of dopa was 0·001. When this work was first started concentrations of 0·02 per cent of dopa and tyrosine were used. It has been found advantageous to reduce these until the present level of 0·001 per cent has been reached, as these lower concentrations prolong the period during which melanin formation can be estimated by the colorimeter. All the solutions inhibited the tyrosine-tyrosinase reaction, no significant difference being observed between the different carbamates. But when the effects on the autoxidation of dopa were investigated, it was found that ethyl carbamate produced a greater degree of acceleration than any of the others. The concentrations used were methyl carbamate 4·000 per cent, ethyl carbamate 4·747 per cent, propyl carbamate 5·494 per cent, butyl carbamate 6·240 per cent.

TABLE V.—*Autoxidation of Dopa in the Presence of Methyl, Ethyl, Propyl and Butyl Carbamates in Equimolecular Proportions* (25.v.50).

| | | *Increments of "E."* | | | |
|---|---|---|---|---|---|
| Carbamate :— Time. | None. | Methyl. | Ethyl. | Propyl. | Butyl. |
| 60 | ·0450 | ·0500 | ·0557 | ·0508 | ·0554 |
| 120 | ·1242 | ·1332 | ·1475 | ·1357 | ·1435 |
| 180 | ·2063 | ·2211 | ·2396 | ·2218 | ·2303 |
| 240 | ·2855 | ·3078 | ·3316 | ·3056 | ·3195 |
| 300 | ·3613 | ·3879 | ·4177 | ·3866 | ·4083 |

It therefore appears that the difference in the action of these carbamates is not in their effect on enzymatic oxidation, but on autoxidation. The difference, however, is not great, and certainly does not seem to be of sufficient magnitude to allow of any explanation of the known action on leukaemia. It was thought possible that the effect of another water-soluble carcinogen and chemotherapeutic agent might throw further light on the problem. The effect of di(2-chloroethyl) methylamine hydrochloride on the oxidation of tyrosine was investigated. The nitrogen mustard was used in strengths up to 0·2 per cent, and at this concentration had no effect whatever on the enzymatic oxidation of tyrosine. The effect on the autoxidation of dopa was then investigated. The lowest concentration used was 0·05 per cent, and at this strength a marked effect was produced. For approximately the first 2 hours the oxidation in the solution containing the nitrogen mustard was accelerated ; for the second 2 hours retarded. The experiment was performed 8 times, with the same result, thus establishing statistical significance. For the last of these experiments readings were taken every 15 minutes until the change from acceleration to retardation occurred. It is of particular interest to compare the differences between these two solutions, when it will be noticed that the changes are, within the limits of accuracy of the method, very regular.

TABLE VI.—*Effect of Di(2-chloroethyl) Methylamine Hydrochloride on the Autoxidation of Dopa* (13.iv.50).

Solution A, control. Solution B, plus nitrogen mustard.

| | *Increments of "E."* | | |
|---|---|---|---|
| Time. | Solution A. | Solution B. | B—A. |
| 15 | ·0066 | ·0089 | ·0023 |
| 30 | ·0133 | ·0204 | ·0071 |
| 45 | ·0271 | ·0322 | ·0051 |
| 60 | ·0414 | ·0443 | ·0029 |
| 75 | ·0586 | ·0517 | —·0069 |
| 90 | ·0791 | ·0618 | —·0172 |
| 120 | ·1234 | ·0774 | —·0260 |
| 180 | ·2174 | ·1019 | —·1155 |
| 240 | ·3099 | ·1220 | —·1879 |

The suggested explanation in this case is that an intermediate metabolite of the interaction between nitrogen mustard and dopa gradually accumulates until it reaches such a concentration that it inhibits the reaction, thus masking the accelerating effect of the nitrogen mustard.

It therefore appears that there is a similarity of action between di(2-chloroethyl) methylamine hydrochloride and the urethanes, and that this is in their action on autoxidation and not on enzymatic oxidation. It is obvious that it would be quite unjustifiable to attempt to explain the action of these substances in the leukaemias and other reticuloses on the grounds of their effects on melanin formation. On the other hand, evidence is produced for one action in which these substances of greatly different chemical constitution behave in a similar manner. In view of our present lack of knowledge of the reticuloses, and especially because of their almost inevitably fatal nature, it might be of considerable interest and possibly of value to investigate the effect on these diseases of other groups of substances which accelerate the progress of autoxidations. The author has been unable to obtain any information on such substances.

### SUMMARY.

Ethyl carbamate inhibits the enzymatic oxidation of tyrosine, but accelerates the autoxidation of dopa. It has at first an inhibiting and later an accelerating action on the enzymatic oxidation of dopa.

Methyl, propyl and butyl carbamates have the same effects, but to a lesser degree.

The water-soluble nitrogen mustard, di(2-chloroethyl) methylamine hydrochloride does not inhibit the enzymatic oxidation of tyrosine in a concentration of 0·2 per cent. It first accelerates and then retards the autoxidation of dopa.

Suggestions for the explanation of these findings are made.

It is suggested that other substances affecting autoxidations in a similar manner might produce effects in the reticuloses.

The work has been carried out with the aid of a grant from the Government Grant Committee of the Royal Society.

### REFERENCES.

HADDOW, A., AND SEXTON, W.—(1946) *Nature*, **157**, 500.

RAPER, H. S.—(1926) *Biochem. J.*, **20**, 735.